# Apolipoprotein E3 Inhibits Rho to Regulate the Mechanosensitive Expression of Cox2

**DOI:** 10.1371/journal.pone.0128974

**Published:** 2015-06-11

**Authors:** Bernadette Y. Hsu, Yong Ho Bae, Keeley L. Mui, Shu-Lin Liu, Richard K. Assoian

**Affiliations:** 1 Program in Translational Biomechanics, Institute of Translational Medicine and Therapeutics, University of Pennsylvania, Philadelphia, Pennsylvania, United States of America; 2 Department of Systems Pharmacology and Translational Therapeutics, University of Pennsylvania, Philadelphia, Pennsylvania, United States of America; University of California, Berkeley, UNITED STATES

## Abstract

Apolipoprotein E3 (apoE3) is thought to protect against atherosclerosis by enhancing reverse cholesterol transport. However, apoE3 also has cholesterol-independent effects that contribute to its anti-atherogenic properties. These include altering extracellular matrix protein synthesis and inhibiting vascular smooth muscle cell proliferation. Both of these cholesterol-independent effects result from an apoE3-mediated induction of cyclooxygenase-2 (Cox2). Nevertheless, how apoE3 regulates Cox2 remains unknown. Here, we show that apoE3 inhibits the activation of Rho, which reduces the formation of actin stress fibers and focal adhesions and results in cellular softening. Inhibition of Rho-Rho kinase signaling or direct cellular softening recapitulates the effect of apoE3 on Cox2 expression while a constitutively active Rho mutant overrides the apoE3 effect on both intracellular stiffness and Cox2. Thus, our results describe a previously unidentified mechanism by which an atheroprotective apolipoprotein uses Rho to control cellular mechanics and Cox2.

## Introduction

Many cell types live in elastic microenvironments and respond to changes in the stiffness of their microenvironments by altering their proliferation, migration and survival [1–4]. Changes in microenvironmental stiffness are typically associated with remodeling of the extracellular matrix (ECM); ECM stiffness is transduced into intracellular stiffness and stiffness-dependent signaling in a process called mechanotransduction [1–3]. ECM-coated hydrogels of distinct elastic moduli have been used to study the signaling events that respond to changes in ECM stiffness [1–5]. Results from this work show that Rho-family GTPases play critical roles in transducing ECM stiffness into intracellular stiffness [1–3]. ECM stiffness stimulates Rho-GTP activity and Rho-Rho kinase (ROCK)-myosin signaling [3–5]. This ultimately leads to acto-myosin-dependent contraction and increases in intracellular tension and formation of actin stress fibers and focal adhesions [6–8]. Focal adhesions are rich in kinases, GTPases, phosphatases, scaffolding proteins, and other signaling molecules that control proliferation, migration and differentiation in several cell types.

Apolipoprotein E (apoE) is a component of triglyceride-rich lipoproteins and plays a major role in limiting atherosclerosis. Humans have three forms of apoE (apoE2, E3 and E4); apoE3 is considered the parent form of the molecule [9]. When associated with high-density lipoprotein (HDL), apoE3 stimulates the transport of cholesterol from peripheral tissues to the liver in a process termed reverse cholesterol transport [10,11]. However, apoE3 is dissociable from HDL in vivo [12], and previous reports have suggested that free apoE3 has cholesterol-independent effects that also contribute to cardiovascular protection [13–16]. We have previously reported that apoE3 inhibits the proliferation of vascular smooth muscle cells (VSMCs) by increasing the levels of the cdk inhibitor, p27^kip1^ [17]. ApoE3 also represses the expression of several ECM proteins associated with arterial stiffening [18], a cholesterol-independent risk factor for a first cardiovascular event [19].

The anti-proliferative and ECM-remodeling effects of apoE3 reflect the stimulatory effect of apoE3 on cyclooxygenase-2 (Cox2) mRNA and protein [15,18]. Cox2 is a major therapeutic target of nonsteroidal anti-inflammatory drugs and plays a key role in cardiovascular biology [20]. In VSMCs, an increase in Cox2 enhances production of PGI_2_, which is a proximal regulator of p27^kip1^ levels and ECM remodeling [17,18]. Importantly, the effects of apoE3 on Cox2, p27^kip1^, and the ECM are independent of the apoE3 lipid-binding domain [17,18], consistent with the notion that these effects are independent of the established apoE3 effect on reverse cholesterol transport. However, the mechanism by which apoE3 regulates Cox2 is unknown.

Here, we have linked the apoE3-mediated regulation of Cox2 to an inhibitory effect of apoE3 on Rho-GTP and intracellular stiffness. We show that apoE3 inhibits the activation of Rho and that this effect reduces the formation of actin stress fibers and focal adhesions to result in cellular softening. Direct inhibition of Rho or cellular stiffness recapitulates the effect of apoE3 on Cox2 while constitutive activation of Rho blocks Cox2 induction in apoE3-treated human VSMCs. Thus, our results describe a previously unidentified mechanism by which apoE3 uses Rho to regulate intracellular mechanics, up-regulate Cox2, and initiate the diverse effects of apoE3 on cellular function.

## Materials and Methods

### Human vascular smooth muscle cell culture

Human aortic vascular smooth muscle cells were purchased from LONZA or ATCC and were grown in Dulbecco’s modified Eagle’s medium (DMEM, 0.5 mg/ml gentamicin, 1 mM sodium pyruvate) containing 10% fetal bovine serum (FBS) until 80–90% confluence. For experiments, cells were incubated in fresh medium with 10% FBS in the absence or presence of 30 μM Y27632 (Calbiochem), 0.03 μM jasplakinolide (Calbiochem), 0.5 μM latrunculin B (Calbiochem), or 2 μM apoE3 for selected times up to 24 hr. This concentration of apoE3 is in the physiological range and regulates both cell cycling and ECM remodeling [18]. For the Rho-GTP assays, near confluent cells were serum-starved for 48 hr in serum-free DMEM with heat-inactivated fatty acid-free bovine serum albumin (BSA; 1 mg/ml). In some experiments, cells in 10% FBS were incubated on 18-mm or 40-mm fibronectin-coated polyacrylamide hydrogels set to the stiffness of healthy or diseased arteries (2–4 and 20–25 kPa, respectively; [2,18,21]). For adenoviral infections, the VSMCs were infected with different adenoviruses overnight at the following multiplicities (MOI): LacZ, 30 or 300; Rho^V14^, 30; Rho^N19^, 300. Media was replaced with fresh culture medium with 10% FBS the next morning immediately prior to addition of apoE3.

### ApoE3 dialysis and protein quantification

Human recombinant apoE3 was dialyzed overnight at 4°C in PBS using a 10,000 MWCO Slide-A-Lyzer Dialysis Cassette from Thermo-Scientific. ApoE3 protein concentrations were determined spectrophotometrically. In some experiments, human recombinant apoE3 was purchased from Sigma.

### Atomic force microscopy (AFM)

Intracellular stiffness was measured by plating cells on glass coverslips or 18-mm fibronectin-coated low- or high-stiffness polyacrylamide hydrogels (2–4 and 20–25 kPa, respectively) in 10% FBS-containing culture media overnight. AFM in force mode was applied to single adherent cells using a DAFM-2X Bioscope (Veeco) mounted on an Axiovert 100 microscope (Zeiss). Cells were indented with a conical tip (40 nm in diameter) against a standard silicon nitride cantilever (spring constant = 0.06 N/m). Intracellular stiffness (Elastic modulus) was quantified by fitting the first 600 nm of tip deflection from the horizontal with the Hertz model for a cone. The tip was positioned near the edge of the cell to measure intracellular stiffness. Measurements were collected for 7–10 cells per condition, and the data were analyzed using custom MATLAB scripts generously provided by Paul Janmey.

### Fluorescence microscopy

Human VSMCs were grown in a 24-well plate on glass coverslips to 70% confluence before stimulation with fresh DMEM-10% FBS in the absence or presence of apoE3 for 24 hr. Coverslips were fixed with formaldehyde and stained with a 1:200 dilution of anti-paxillin (Santa Cruz Biotechnology) or anti-vinculin (Sigma). Cells were also stained with a 1:500 dilution of FITC-conjugated phalloidin (Alexa Fluor 594, Life technologies).

### Rho GTPase Assay

Human VSMCs were cultured in a 35-mm tissue culture dish until 80–90% confluent and then serum-starved in BSA-containing DMEM for 48 hr. The cells were then stimulated with 10% FBS in the presence or absence of 2 μM apoE3 for 9 hr. A similar protocol was used to measure the effect of apoE3 on Rho-GTP levels in human VSMCs plated on fibronectin-coated low- or high-stiffness hydrogels for 24 hr, 2–4 kPa and 20–25 kPa respectively [2]. Active Rho GTPase levels were measured using a G-LISA small G-protein activation assay kit (Cytoskeleton) according to the manufacturer’s directions.

### Statistical analysis

Data are presented as mean + standard error of mean (SEM) of the indicated number of independent experiments. Data were analyzed with a nonparametric Mann-Whitney test or t-test. Results with *p*-values lower than 0.05 (*), 0.01 (**), or 0.001 (***) were considered to be statistically significant.

## Results

### ApoE3 controls cell mechanics by inhibiting Rho-GTP

**Fig 1:**
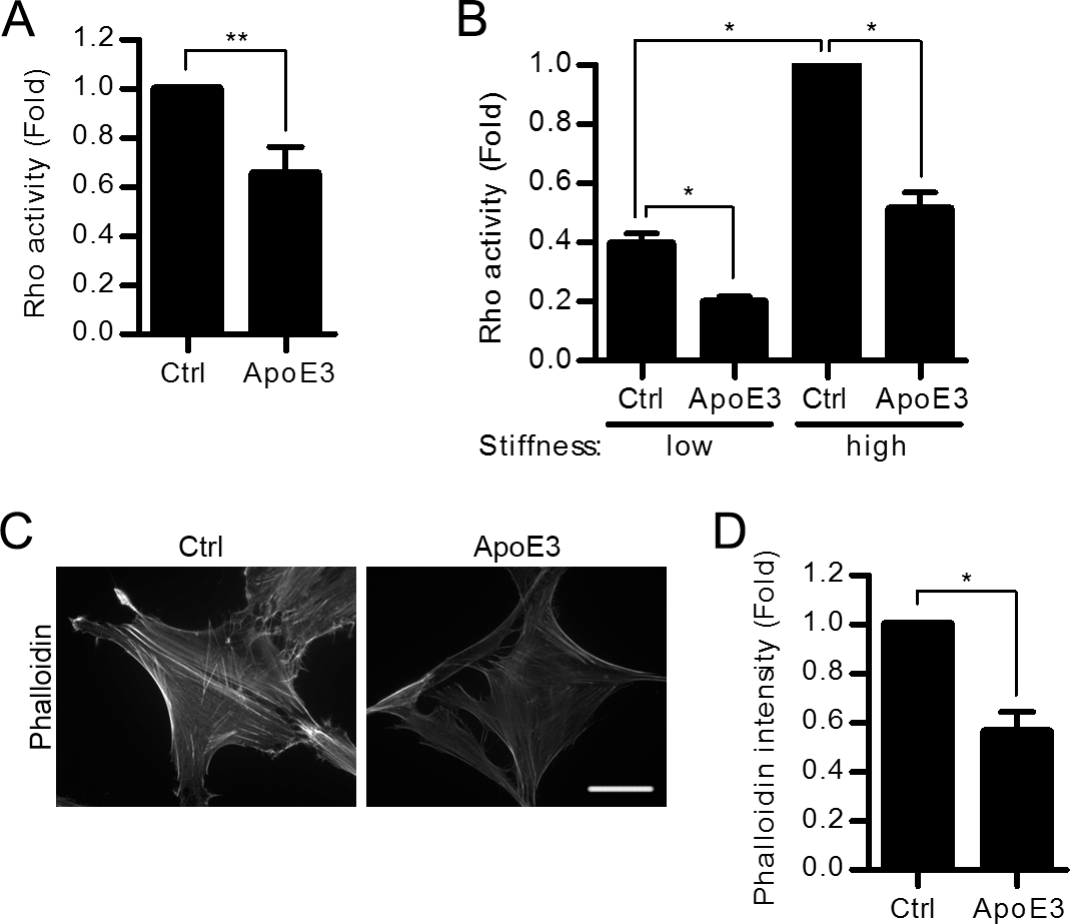
ApoE3 decreases Rho activity and F-actin stress fibers in human VSMCs. (A-B) Starved VSMCs were incubated with 10% FBS in a culture dish (A) or on fibronectin-coated low or high stiffness hydrogels (B) with or without apoE3 treatment for 9 hr. Rho activity was measured. *n = 4*. (C) VSMCs were incubated with 10% FBS in the absence or presence of apoE3 for 24 hr. Scale bar = 50 μm. Fixed cells were incubated with FITC-conjugated phalloidin to stain f-actin. (D) Average phalloidin intensity of single cells was analyzed using ImageJ and normalized to control cells. *n = 4* independent experiments with 5–10 cells analyzed per experiment. Data information: Graphs show mean + SEM. *p<0.05 or **p<0.01.

Our previous work used fibronectin-coated hydrogels set to physiologic and pathologic arterial stiffness to show that Cox2 levels in VSMCs are repressed by stiff substrata and that apoE3 overrides this effect to increase Cox2 expression on stiff surfaces [18]. Since ECM stiffness is transduced into the cell by signaling through the Rho-ROCK pathway (see Introduction), we reasoned that the apoE3 effect on Cox2 might reflect regulation of Rho signaling. Indeed we found that Rho-GTP levels were significantly reduced by apoE3 in human VSMCs (Fig 1A). ApoE3-mediated inhibition of Rho activity was also detected when the VSMCs were cultured at pathophysiologically relevant ECM stiffness on fibronectin-coated polyacrylamide hydrogels matched to the stiffness of healthy and atherosclerotic arteries (Fig 1B and [22]).

**Fig 2:**
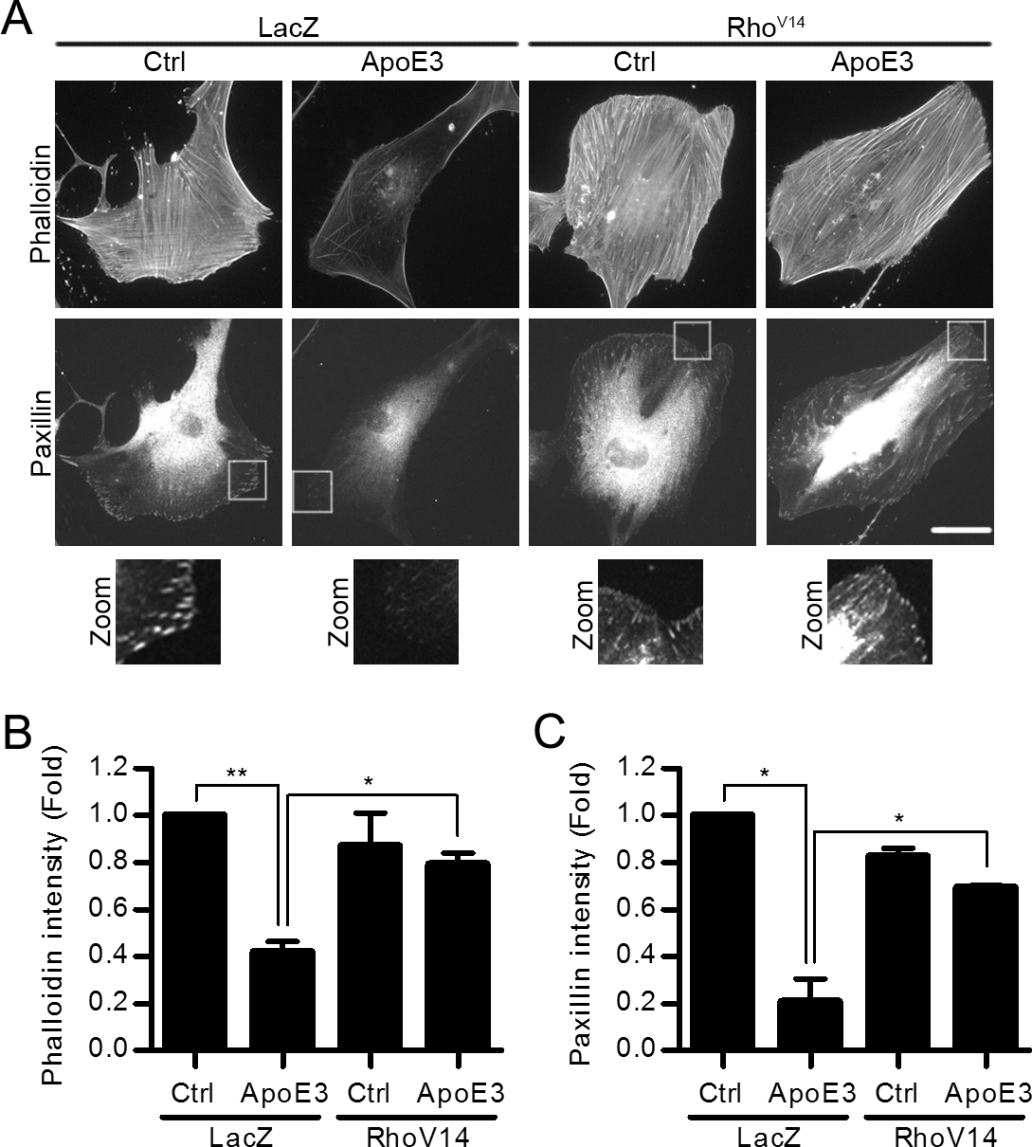
Constitutively active Rho rescues actin stress fibers and paxillin in the presence of apoE3. (A) VSMCs infected with adenoviruses encoding LacZ or Rho^V14^ were incubated in 10% FBS with or without apoE3 for 24 hr. Cells were co-stained with phalloidin and anti-paxillin. Scale bar = 50 μm. The zooms show magnified views of paxillin staining in the boxed areas. The average phalloidin (B) and paxillin (C) intensity of single cells was analyzed using ImageJ and normalized to control cells. *n = 3* independent experiments with at least 8 cells analyzed per experiment. Data information: Graphs show mean + SEM. *p<0.05 or **p<0.01.

The Rho-ROCK signaling pathway regulates acto-myosin-dependent contraction, intracellular tension, and the formation of actin stress fibers and associated focal adhesions [6–8]. Consistent with the inhibitory effect of apoE3 on Rho activity, f-actin stress fiber intensity (Figs 1C and 1D and 2A and 2B; LacZ) was reduced in VSMCs treated with apoE3. Focal adhesion abundance and areas were also reduced as determined by staining for either paxillin (Fig 2A and 2C; LacZ and S1A, S1B and S1C Fig) or vinculin (S1D and S1E Fig). The levels of total paxillin (S1F Fig) and vinculin (S1G Fig) were unchanged in apoE3-treated cells compared to controls. Thus, inhibition of intracellular stiffness by apoE3 affects the distribution rather than total quantity of focal adhesion proteins. These effects closely resembled those seen with dominant negative Rho (Rho^N19^, S2A Fig) or a selective ROCK inhibitor (Y27632, S2B Fig).

**Fig 3:**
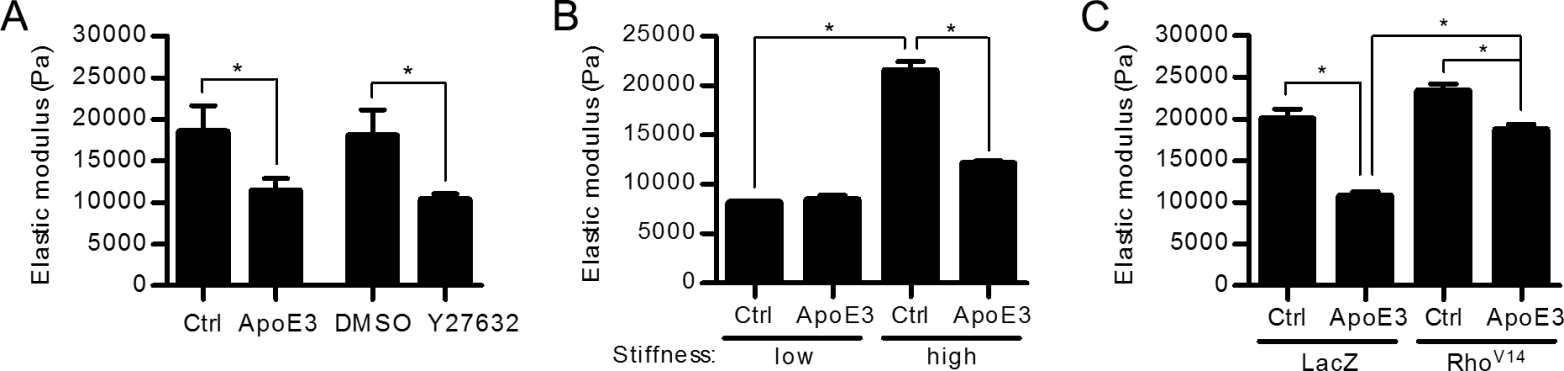
ApoE3 reduces intracellular stiffness through the Rho signaling pathway. (A) VSMCs in 10% FBS were treated with apoE3 or Y27632, or their respective vehicle controls (Ctrl), for 24 hr. Intracellular stiffness was determined by AFM. *n = 4*. (B) VSMCs were plated on fibronectin-coated low or high stiffness hydrogels in 10% FBS with or without apoE3 for 24 hr, and intracellular stiffness was determined by AFM. *n = 4*. (C) VSMCs infected with adenoviruses encoding LacZ or Rho^V14^ were incubated with 10% FBS in the absence or presence of apoE3 for 24 hr, and intracellular stiffness was measured by AFM. *n = 4*. Data information: Graphs show mean + SEM. *p<0.05.

Actin stress fibers and focal adhesions are canonical indicators of intracellular stiffness, and indeed we found that apoE3 reduced intracellular stiffness as determined by AFM of human VSMCs cultured on both rigid (Fig 3A, columns 1 and 2) and pathophysiologically relevant (Fig 3B) substrata. Moreover, this effect of apoE3 was similar in magnitude to that seen with Y27632 (Fig 3A, columns 3 and 4). Importantly, these effects of apoE3 and Rho-ROCK signaling are causally related because ectopic expression of an activated Rho (Rho^V14^) rescued actin stress fibers (Fig 2A; phalloidin staining; Rho^V14^), focal adhesions (Fig 2A; paxillin staining; Rho^V14^), and intracellular stiffness (Fig 3C) in the presence of apoE3.

### ApoE3-mediated inhibition of Rho-GTP regulates the expression of Cox2

**Fig 4:**
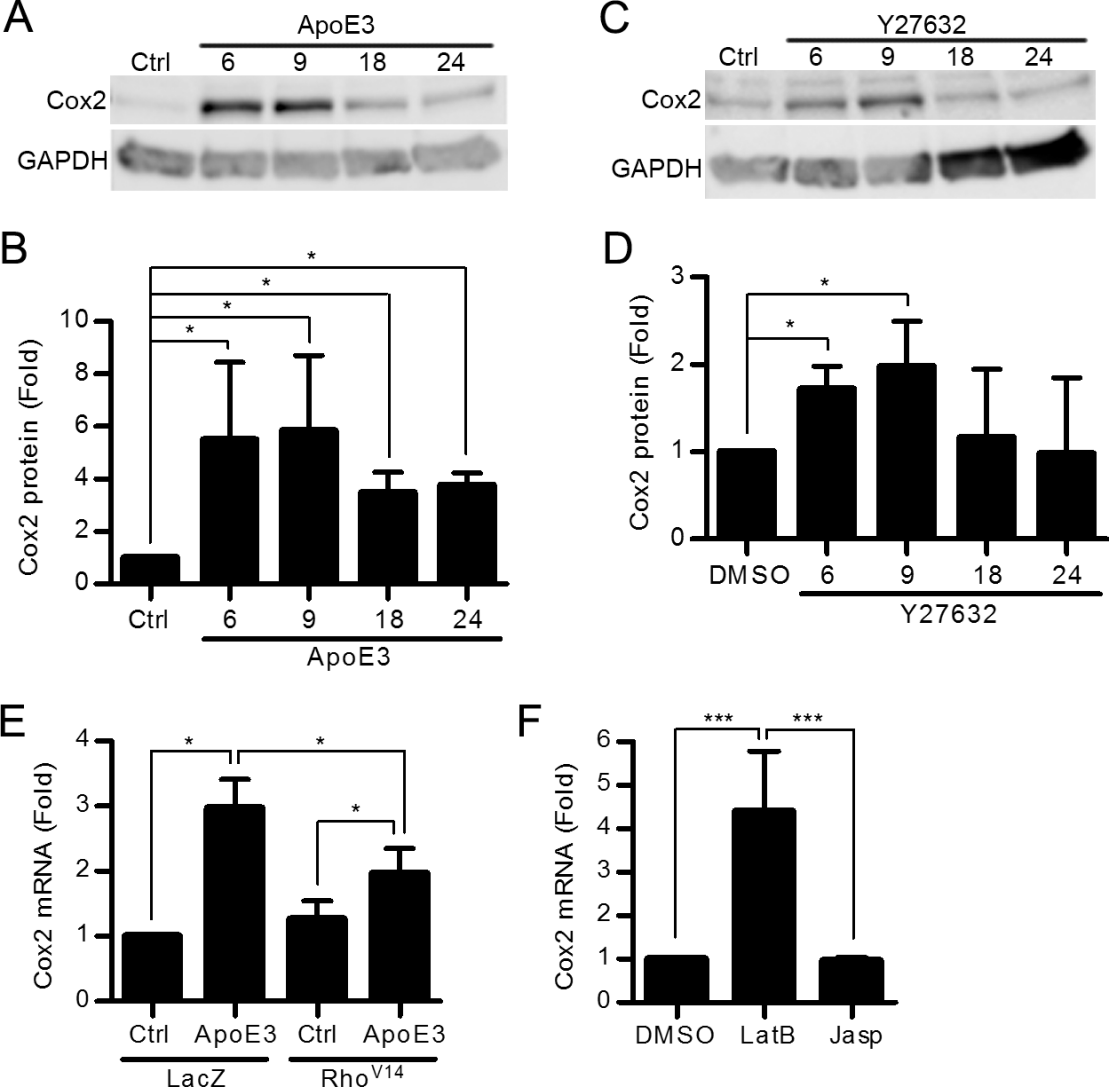
Inhibition of Rho/ROCK signaling mimics the stimulatory effect of apoE3 on Cox2 expression. (A-E) Human VSMCs in 10% FBS were treated with apoE3 (A) or Y27632 for the times shown (C). Total cell lysates were immunoblotted for Cox2 and GAPDH (A and C). The bar graphs show Cox2 levels normalized to control (B and D). *n = 4*. In E, Cox2 mRNA was quantified by RT-qPCR and plotted relative to 18S rRNA. *n = 4*. (F) VSMCs in 10% FBS were treated with vehicle (DMSO), latrunculin B (LatB), or jasplakinolide (Jasp) for 6 hr. Cox2 mRNA was determined by RT-qPCR; *n* = 4. Data information: Graphs show mean + SEM. *p<0.05 or ***p<0.001.

To determine if the effect of apoE3 on Cox2 gene and protein expression might be a consequence of its ability to inhibit the Rho signaling pathway, human VSMCs were treated with apoE3 or Y27632. Both of these treatments increased Cox2 protein levels by 6–9 hr (Fig 4A, 4B, 4C and 4D). Cox2 levels then declined with both treatments although the level of Cox2 returned to that of untreated controls in the VSMCs treated with Y27632 whereas it remained elevated relative to the control cells for at least 24 hr in VSMCs treated with apoE3 (Fig 4A, 4B, 4C and 4D). We reasoned that this difference might reflect the fact that apoE3 inhibits all of Rho signaling while Y27632 only inhibits the Rho kinase effector pathway. Consistent with this notion, we found that Cox2 levels remained elevated in VSMCs for at least 24 hr after infection with dominant negative Rho (S3A and S3B Fig).

The effects of apoE3 and Rho/ROCK inhibition on Cox2 protein levels were also seen when examining the level of Cox2 mRNA (S4A, S4B and S4C Fig). Moreover, expression of constitutively active Rho partially overcame the stimulatory effect of apoE3 on Cox2 gene expression (Fig 4E). This finding demonstrates that an apoE3-mediated inhibition of Rho-GTP is causal for the stimulatory effect of apoE3 on Cox2 expression.

If the inhibitory effect of apoE3 on Rho-dependent actin organization and intracellular stiffness accounts for its stimulatory effect on Cox2 mRNA, then direct manipulation of the actin cytoskeleton should recapitulate the effect of apoE3 on Cox2 levels. We therefore treated human VSMCs with two pharmacologic regulators of actin polymerization. Latrunculin B, which inhibits actin polymerization by binding actin monomers [21–24] and reduces intracellular stiffness [1,22,24], mimicked the effect of apoE3 by increasing Cox2 mRNA levels (Fig 4F). In contrast, jasplakinolide, which promotes actin nucleation, stabilizes stress fibers [25], and increases intracellular stiffness [1], was without effect (Fig 4F). Collectively, these results indicate that the reduction in intracellular stiffness occurring in response to Rho inhibition leads to the increase in abundance of Cox2.

## Discussion

**Fig 5:**
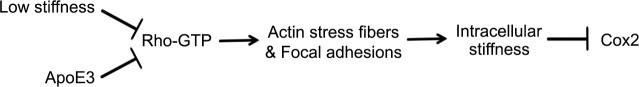
ApoE3 inhibits Rho-GTP to regulate Cox2. A working model depicting how apoE3 regulates Rho and intracellular stiffness and how these effects regulate mechanosensitive Cox2 expression.

We show here that apoE3 is a potent inhibitor of Rho-GTP activity in VSMCs and that direct inhibition of Rho signaling phenocopies the effect of apoE3 on Cox2 mRNA and protein expression. Moreover, ectopic expression of activated Rho rescues stress fiber and focal adhesion formation (Fig 2A, 2B and 2C), cellular stiffness (Fig 3C), and Cox2 expression (Fig 4E) in apoE3-treated VSMCs. These finding demonstrate that the effect of apoE3 on Rho-GTP is causal for the apoE3 effects on cellular stiffness and Cox2 gene expression (Fig 5).

Inhibition of Rho signaling reduces intracellular stiffness, and our data with latrunculin D, which phenocopies the effect of apoE3 on intracellular stiffness and Cox2, link the increase in Cox2 gene expression to decreased intracellular stiffness itself. Intracellular stiffness affects f-actin/g-actin ratios, which are known to control gene transcription through YAP/TAZ and MRTF transcriptional regulators [26]. However, reduced stiffness and f-actin/g-actin ratios should also attenuate signaling through integrins and associated focal adhesion complexes. The mechanistic basis of apoE3- and Rho-regulated expression of Cox2 mRNA is an important matter for future study.

Others have examined the relationship between Rho-ROCK signaling, the actin cytoskeleton, and apolipoprotein(a), a distinct apolipoprotein that is pro- (rather than anti-) atherogenic [27]. Apolipoprotein(a) rapidly increases Rho-GTP levels and actin stress fibers in endothelial cells and VSMCs [28,29]. Thus, apolipoprotein(a) and apoE3 have opposing effects on Rho-GTP that correspond to their opposing effects on atherosclerosis. These results raise the possibility that pharmacologic inhibition of VSMC Rho-GTP might limit atherosclerosis by increasing Cox2 and the production of cardiovascular protective prostanoids such as PGI_2_ [18].

We recently reported that the expression of collagen-I and lysl oxidase as well as arterial stiffness are elevated in apoE-null mice relative to wild-type controls. Moreover, we could show that increased arterial stiffening was causal for lesion formation because pharmacologic inhibition of collagen-I crosslinking in apoE-null mice reduced arterial stiffness and atherosclerosis in apoE-null mice despite elevated cholesterol [18]. Since cells alter their internal stiffness in response to changes in the stiffness of the ECM [1,23,25], VSMCs in the (stiff) atherosclerotic aorta are likely abnormally stiff themselves. We propose that apoE3 attenuates this intracellular stiffening of VSMCs by limiting Rho-GTP activity.

Many studies have identified reverse cholesterol transport as a mechanism by which apoE3 provides cardiovascular protection [10,11]. However, the effects of apoE3 extend beyond regulation of plasma lipid levels. Our work shows that apoE3 also controls cell mechanics by inhibiting the Rho/ROCK signaling pathway to reduce intracellular stiffness and override stiffness-dependent downregulation of Cox2. These effects likely limits SMC proliferation, ECM remodeling, and arterial stiffening [17], all of which contribute to cardiovascular disease.

## Supporting Information

S1 File Extended Experimental Procedures. (PDF)

S1 Fig ApoE3 inhibits paxillin and vinculin localization to the focal adhesions. (PDF)

S2 Fig Effect of Rho-ROCK inhibition on actin stress fibers and paxillin-containing focal adhesions. (PDF)

S3 Fig Rho inhibition increases Cox2 expression. (PDF)

S4 Fig Inhibition of Rho/ROCK signaling mimics the stimulatory effect of apoE3 on Cox2 expression. (PDF)
