# Supplementary material for: Apolipoprotein E3 Inhibits Rho to Regulate the Mechanosensitive Expression of Cox2
Source: PLoS One. 2015 Jun 11;10(6):e0128974. doi: 10.1371/journal.pone.0128974 (PMC4465925; doi:10.1371/journal.pone.0128974)
Supplement: S1 File — (PDF) [file pone.0128974.s001.pdf]

Extended Experimental Procedures

Immunoblotting

Human VSMCs were lysed in TNE buffer (50mM Tris-HCl, pH 8; 250 mM NaCl; 2 mM EDTA; 1% Non-idet P-40) and total protein concentration was determined using Protein Assay Dye Reagent Concentrate (Bio-Rad). Equal amounts of protein (10-50 µg) were fractionated and analyzed by immunoblotting using antibodies specific for Cox2 (Cayman Chemical), paxillin (Santa Cruz Biotechnology), vinculin (Sigma) or GAPDH (Santa Cruz Biotechnology). Fractionated proteins were visualized by enhanced chemiluminescence, and band intensities were measured using the gel analyzer package in ImageJ.

Immunofluorescence microscopy

Coverslips were washed once with PBS before fixation with 3.7% formaldehyde in PBS. Cells were then permeabilized with 0.4% Triton X-100 in PBS for 15 min at room temperature, blocked with a solution of 0.2% Triton-X 100 and 2% BSA in PBS for 1 hr at room temperature, and incubated with primary antibodies for 1 hr at room temperature or overnight at 4°C. After the primary antibody incubation, the cells were washed 3 times with blocking solution for 5 min each and then incubated with (1:200 dilution) rhodamine (TRITC)-AffiniPure F(ab')2 rabbit anti-goat (Invitrogen) or (1:200 dilution) Alexa Fluor 488-conjugated chicken anti-rabbit (Invitrogen) for 1 hr at room temperature. Following the secondary antibody incubation, the cells were washed 3 times with blocking solution for 5 min each and then once with distilled water. Coverslips were mounted with *SlowFade* Gold antifade reagent (Invitrogen) on glass slides. Images were digitally acquired using a Hamamatsu camera at 20X and 40X magnification.

Image Analysis

Focal adhesion intensities were quantified using ImageJ. Regions of interest were defined using the polygon selection tool, and the raw integrated density within the region of interest was calculated using the measurement tool. Background raw integrated densities were subtracted from the raw integrated densities, and the resulting number was then normalized to the total measured area.

Representative images from several independent experiments were analyzed in Image J to determine the intensity of stained actin stress fibers. A line perpendicular to the stress fibers and avoiding the nucleus was drawn across each cell, and a plot profile was obtained. The distance and corresponding gray scale numbers were copied and graphed in Microsoft Excel. Background signal intensity was subtracted within Excel by fitting a trend line (linear regression) to the distance vs. gray scale graph. All numbers less than or equal to zero were changed to 0, and a Riemann sum was used to obtain the area under the intensity plot. Finally, the area was normalized to the length of the drawn line and this area/distance ratio was used to compute total actin intensities in control and apoE3-treated cells.

RT-qPCR

Human VSMCs were grown to 80-90% confluence on 12-mm glass coverslips in a 24-well plate or 12-well plastic cell culture plates. Cells were stimulated with 10% FBS in the presence or absence of apoE3, DMSO, jasplakinolide, latrunculin B, or Y27632 for selected times up to 24 hr. The cells were lysed in 0.5 ml Trizol reagent (Invitrogen). Total RNA was isolated using standard procedures, and a small aliquot of RNA was quantified spectrophotometrically at 260 nm. The ratio of absorbance at 260 and 280 nm was measured to ensure RNA purity. RNA (50 ng) from each sample was used for reverse transcription into cDNA with Applied Biosystems

reagents (1× buffer, 5.5 mM $MgCl_2$, 500 µM of each dNTP, 2.5 µM random hexamers, 0.4 U/ml RNase inhibitor, and 1.25 U/ml Multiscribe Reverse Transcriptase) in a total volume of 20 µl. Real-time PCR reactions were then performed with 2 µl of the cDNA solution, 1x TaqMan Universal PCR mix (Applied Biosystems), and the appropriate TaqMan Assay on Demand (for human Cox2 mRNA) or forward and reverse primers and probe for 18S rRNA as described by Klein et al., 2007 [1]. The solution was brought to a final volume of 20 µl with water, and the reaction was performed using an Applied Biosystems Prism 7000 sequence detection system. Data were plotted using either the standard curve or delta delta CT methods.

References

1. Klein EA, Yung Y, Castagnino P, Kothapalli D, Assoian RK (2007) Cell Adhesion, Cellular Tension, and Cell Cycle Control. In: David AC, editor. Methods in Enzymology: Academic Press. pp. 155-175.
