# Supplementary material for: Apolipoprotein E3 Inhibits Rho to Regulate the Mechanosensitive Expression of Cox2
Source: PLoS One. 2015 Jun 11;10(6):e0128974. doi: 10.1371/journal.pone.0128974 (PMC4465925; doi:10.1371/journal.pone.0128974)
Supplement: S1 Fig — (PDF) [file pone.0128974.s002.pdf]

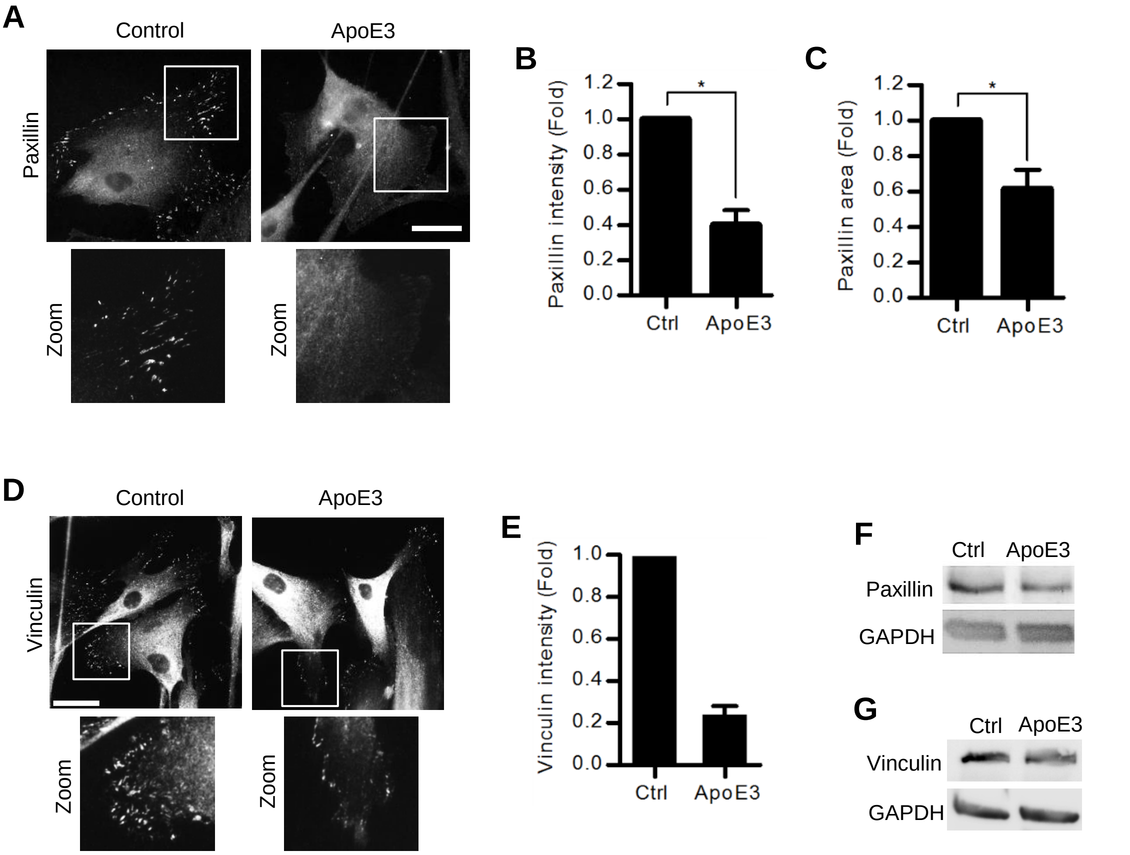

S1 Fig. ApoE3 inhibits paxillin and vinculin localization to the focal adhesions. Human VSMCs were incubated with 10% FBS in the absence or presence of apoE3 for 24 hr. Cells were immunostained for paxillin (A) or vinculin (D). Scale bar = 50 µm. Average paxillin (B), or vinculin (E) signal intensities of single cells (at least 10 cells per experiment) were analyzed using ImageJ and normalized to control cells. In C, cells were fixed and co-stained with anti-paxillin and phalloidin. The color stack tool in ImageJ was then used to identify paxillin-containing focal adhesions at the ends of stress fibers, and the areas of three randomly selected paxillin-containing focal adhesions were measured per cell using the polygon tracer tool in imageJ. Five cells were analyzed in each of 4 independent experiments. Average areas were normalized to those of the control cells. Total cell lysates were also immunoblotted for paxillin (F) and vinculin (G). A, *n=4*; D and G, *n=2*; F, *n=3.* Data information: Graphs show mean + SEM (B-C) or mean + range (E). *P<0.05
