# Supplementary material for: Apolipoprotein E3 Inhibits Rho to Regulate the Mechanosensitive Expression of Cox2
Source: PLoS One. 2015 Jun 11;10(6):e0128974. doi: 10.1371/journal.pone.0128974 (PMC4465925; doi:10.1371/journal.pone.0128974)
Supplement: S2 Fig — (PDF) [file pone.0128974.s003.pdf]

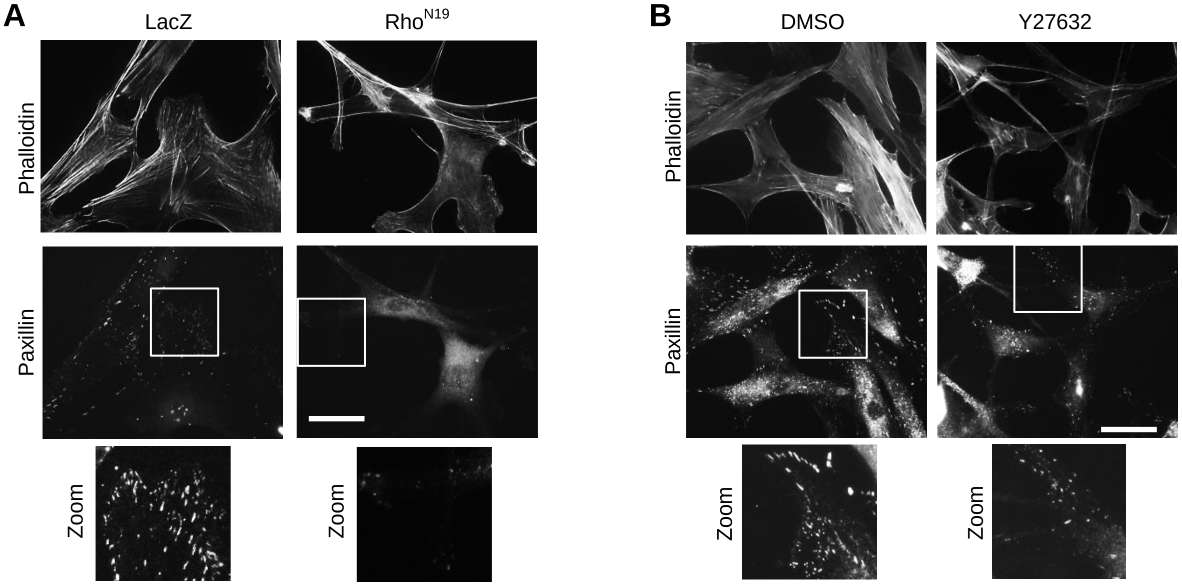

S2 Fig. Effect of Rho-ROCK inhibition on actin stress fibers and paxillin-containing focal adhesions. (A-B) VSMCs infected with adeno-LacZ or adeno-Rho^{N19} (A) or treated with DMSO (vehicle) or Y27632 (B) were incubated in 10% FBS for 24 hr. Cells were fixed, stained with phalloidin or anti-paxillin, and visualized by immunofluorescence microscopy. The zooms show magnified views of paxillin staining in the boxed areas. Scale bar = 50 µm. *n=3*.
