# Supplementary material for: Apolipoprotein E3 Inhibits Rho to Regulate the Mechanosensitive Expression of Cox2
Source: PLoS One. 2015 Jun 11;10(6):e0128974. doi: 10.1371/journal.pone.0128974 (PMC4465925; doi:10.1371/journal.pone.0128974)
Supplement: S3 Fig — (PDF) [file pone.0128974.s004.pdf]

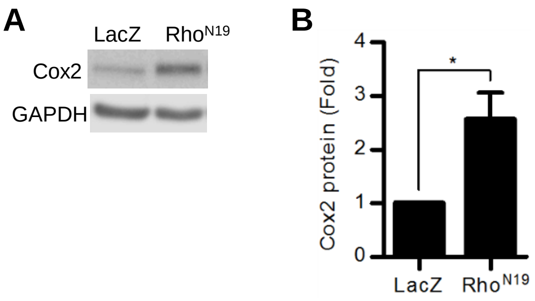

S3 Fig. Rho inhibition increases Cox2 expression. (A) Human VSMCs were infected with adeno-Rho^{N19} for 24 hr and analyzed by immunoblotting. (B) The bar graph shows Cox2 levels normalized to LacZ. Data information: Graph shows mean + SEM. **p*<0.05.
