# Supplementary material for: Apolipoprotein E3 Inhibits Rho to Regulate the Mechanosensitive Expression of Cox2
Source: PLoS One. 2015 Jun 11;10(6):e0128974. doi: 10.1371/journal.pone.0128974 (PMC4465925; doi:10.1371/journal.pone.0128974)
Supplement: S4 Fig — (PDF) [file pone.0128974.s005.pdf]

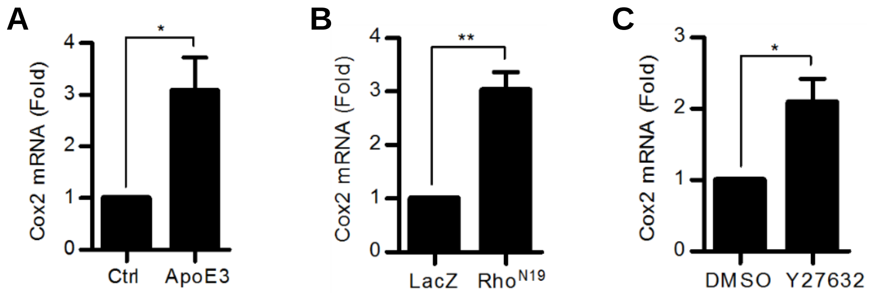

S4 Fig. Inhibition of Rho/ROCK signaling mimics the stimulatory effect of apoE3 on Cox2 expression. Human VSMCs were treated with apoE3 (A), infected with adeno-LacZ or adeno-Rho^{N19} (B), or treated with Y27632 (C). After 24 hr with 10% FBS, Cox2 mRNA was quantified by RT-qPCR and plotted relative to 18S rRNA. $n=4$. Data information: Graphs show mean + SEM. *$p<0.05$ or **$p<0.01$
